# Causes, Consequences and Public Health Implications of Low B-Vitamin Status in Ageing

**DOI:** 10.3390/nu8110725

**Published:** 2016-11-16

**Authors:** Kirsty Porter, Leane Hoey, Catherine F. Hughes, Mary Ward, Helene McNulty

**Affiliations:** Northern Ireland Centre for Food and Health, Ulster University, Cromore Road, Coleraine BT52 1SA, UK; porter-k7@email.ulster.ac.uk (K.P.); l.hoey@ulster.ac.uk (L.H.); c.hughes@ulster.ac.uk (C.F.H.); mw.ward@ulster.ac.uk (M.W.)

**Keywords:** B-vitamins, ageing, degenerative diseases, cardiovascular disease, cognitive dysfunction, dementia, osteoporosis, methylenetetrahydrofolate reductase (MTHFR)

## Abstract

The potential protective roles of folate and the metabolically related B-vitamins (vitamins B12, B6 and riboflavin) in diseases of ageing are of increasing research interest. The most common cause of folate and riboflavin deficiencies in older people is low dietary intake, whereas low B12 status is primarily associated with food-bound malabsorption, while sub-optimal vitamin B6 status is attributed to increased requirements in ageing. Observational evidence links low status of folate and the related B-vitamins (and/or elevated concentrations of homocysteine) with a higher risk of degenerative diseases including cardiovascular disease (CVD), cognitive dysfunction and osteoporosis. Deficient or low status of these B-vitamins alone or in combination with genetic polymorphisms, including the common *MTHFR* 677 C → T polymorphism, could contribute to greater disease risk in ageing by causing perturbations in one carbon metabolism. Moreover, interventions with the relevant B-vitamins to optimise status may have beneficial effects in preventing degenerative diseases. The precise mechanisms are unknown but many have been proposed involving the role of folate and the related B-vitamins as co-factors for one-carbon transfer reactions, which are fundamental for DNA and RNA biosynthesis and the maintenance of methylation reactions. This review will examine the evidence linking folate and related B-vitamins with health and disease in ageing, associated mechanisms and public health implications.

## 1. Introduction

An estimated 900 million people are aged ≥65 years globally, equating with 8% of the world’s population, and by 2050, this is predicted to exceed two billion (16%) [1]. Approximately one quarter of the total global burden of disease is in older people, with a higher prevalence in high income countries [2]. Hypertension, the leading risk factor of cardiovascular disease (CVD) affects an estimated one billion people worldwide and CVD is the most common cause of death in older people [3]. Globally, osteoporotic fractures affect over nine million older people annually [4], while 46.8 million older people are reported to have dementia worldwide [5]. The prevalence of these diseases of ageing is expected to substantially increase as a result of the ever-increasing ageing population. In addition, these degenerative diseases cause multiple co-morbidities in older people which in turn has important societal and economic consequences. Maintaining good health in older age has therefore become a major public health priority. Poor nutrition is recognised as a modifiable risk factor in the development of degenerative diseases in ageing, and improved nutrition may prevent or delay the onset of adverse health outcomes as people age. In this context, the potential adverse effect of elevated homocysteine and/or the protective roles of folate and the metabolically related B-vitamins (B12 and B6), have received much attention. 

This review will examine the emerging evidence linking folate and the metabolically related B-vitamins with ageing, the potential roles of these nutrients in preventing or delaying diseases of ageing and the associated mechanisms. The challenges and opportunities in achieving optimal B-vitamin status in older people will also be considered with particular emphasis on the role of food fortification.

## 2. Metabolic Role of B-Vitamins in One-Carbon Metabolism

**Figure 1:**
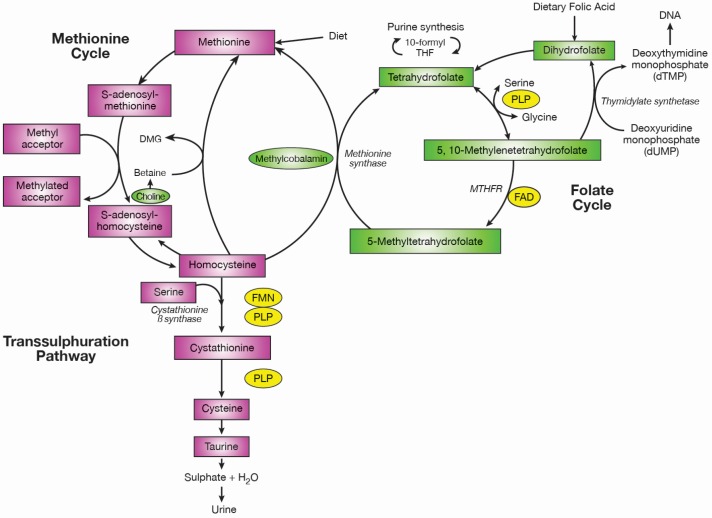
One-carbon metabolism. Abbreviations: PLP, plasma pyridoxal phosphate; MTHFR, methylenetetrahydrofolate reductase; FAD, flavin adenine dinucleotide; FMN, flavin mononucleotide. Adapted from [16].

Folate along with vitamins B12, B6 and riboflavin in their co-enzymatic forms are all essential in one-carbon metabolism (Figure 1), a network of reactions involving the transfer of one-carbon units. In the folate cycle, tetrahydrofolate obtains a carbon unit from serine in a vitamin B6 (plasma pyridoxal phosphate; PLP) dependent reaction forming 5,10-methylenetetrahydrofolate which is used for the synthesis of thymidine and purines or converted to 5-methyltetrahydrofolate. 5-methyltetrahydrofolate is the principal circulating form of folate, and this reaction is catalysed by methylenetetrahydrofolate reductase (MTHFR) using riboflavin (flavin adenine dinucleotide, FAD) as a co-factor. At this point, the folate cycle links with the methionine cycle, 5-methyltetrahydrofolate donates its methyl group to homocysteine for the formation of methionine in a reaction catalysed by methionine synthase which uses vitamin B12 (methylcobalamin) as a cofactor. Methionine is the precursor for *S*-adenosyl-methionine (SAM), the universal methyl donor for DNA and RNA, proteins and numerous central nervous system methylation reactions involving neurotransmitters, membrane phospholipid synthesis and myelin methylation [6,7]. SAM is converted to *S*-adenosylhomocysteine and then homocysteine which is either remethylated back to methionine or conversely metabolised in the transsulphuration pathway to form cysteine through another vitamin B6-dependent process [8]. The metabolism of the B-vitamins is closely related; folate and vitamin B12 are both intrinsically linked via the enzyme methionine synthase [9]. In vitamin B12 depletion, methionine synthase activity is reduced and the formation of tetrahydrofolate is blocked, with folate essentially becoming trapped as 5-methyltetrahydrofolate because the conversion by MTHFR is physiologically irreversible [10]. There is also an important metabolic inter-relationship between vitamin B6 and riboflavin. The conversion of dietary vitamin B6 in tissues to its functional enzyme, pyridoxal 5′ phosphate (PLP), requires the enzyme pyridoxine phosphate oxidase (PPO), which is dependent on the riboflavin in its co-factor form, flavin mononucleotide (FMN).

Deficiencies in any of these B-vitamins can perturb the complex regulatory network maintaining one-carbon metabolism resulting in reduced methylation status within the relevant tissue, hyperhomocysteinemia, and/or increased misincorporation of uracil into DNA as a result of thymidylate synthesis being impaired owing to low 5,10-methylene-THF concentrations and thus uracil is inserted instead during DNA synthesis which in turn may contribute to adverse health outcomes in ageing [11,12]. In addition, genetic polymorphisms, including the common 677 C → T polymorphism in the gene encoding the folate-metabolising enzyme MTHFR, can interact adversely with sub-optimal status of one or more of the B-vitamins in one-carbon metabolism and thus contribute to a greater disease risk [13]. The *MTHFR* 677TT genotype affects an estimated 10% of individuals worldwide (ranging from 3% to 32% depending on ethnicity) [14] and 12% in Ireland [15]. 

## 3. Causes of B-Vitamin Deficiency

Depending on the particular vitamin, there are a number of potential causes of B-vitamin deficiency including inadequate intake, increased requirements, malabsorption, drug–nutrient interactions and others including genetic disorders or medical conditions (Table 1). In addition, the ageing process itself can negatively affect the absorption, transport and metabolism of B-vitamins and thus older people have increased requirements. A recent systematic review in community dwelling older adults in developed Western countries (*n* = 28,000) reported a high prevalence of low dietary intakes for B-vitamins (i.e., below the estimated average requirement, EAR), including folate (29%–35%), vitamin B6 (24%–31%) and identified riboflavin (31%–41%) among six nutrients of potential public health concern [17]. An estimated 9%–12% of older people in the UK are considered to suffer from folate deficiency [18], with the most common cause being low dietary intake.

There is a high prevalence of vitamin B12 deficiency in older people globally [28], with two early studies finding 75% of Asian Indians demonstrating metabolic evidence of vitamin B12 deficiency [29,30]. Numerous population surveys have also identified varying levels of vitamin B12 deficiency and sub-optimal status in older people from the UK (5%–20% deficient) [31,32,33], US (6% deficient, >20% marginal status) [34,35] Canada (5% deficient) [36], New Zealand (12% deficient, 28% marginal deficiency) [37] and Finland (6% low B12 and 32% borderline) [38]. The variation in prevalence may in part be explained by the diagnostic criteria used and different studies have used different biomarkers and cut off points. Pernicious anaemia, the classic form of B12 deficiency, caused by a lack of intrinsic factor is thought to account for just 1%–2% of cases of insufficiency in older people [39]. The maintenance of vitamin B12 status in older people is not only dependent on adequate dietary intake but more critically on the normal absorption of the vitamin which is dependent on the normal functioning of the gastrointestinal tract, gastric acid secretion and a number of transport proteins including intrinsic factor [40]. Food-bound malabsorption, primarily as a result of atrophic gastritis, an age-related disorder affects up to 30% of older people [41], leads to a reduction in gastric acid secretion which prevents the release of B12 from food and thus absorption. Deficiency is less pronounced and the limitations of the conventional vitamin B12 assays may also mean that a significant proportion of the older population may have low vitamin B12 status which is not detected [40,42]. 

Medications including proton pump inhibitors (PPI) and H2-receptor antagonists (H_2_RA) are commonly prescribed (for conditions such as reflux and peptic ulcers) in older people, resulting in gastric acid suppression, this mimics atrophic gastritis and leads to food-bound malabsorption. There is evidence to suggest that these medications have been associated with up to 4.5 times higher risk of vitamin B12 deficiency in case control studies [43,44]. Recently in the USA, a large community survey (25,956 cases and 184,199 controls) found that the long term use (>2 years) of H_2_RAs and PPIs was associated with a 25%–65% greater risk of a subsequent diagnosis of vitamin B12 deficiency [45]. In addition, metformin usage in Type 2 Diabetes can also result in vitamin B12 deficiency, possibly interfering with calcium-dependent membrane action in the terminal ileum required for the absorption of the vitamin B12-intrinsic factor complex [46,47]. 

Vitamin B6 and riboflavin in older people have not been as widely investigated. There is a high prevalence of vitamin B6 deficiency in older people as evident in three large population based surveys in Europe (Survey in Europe Nutrition and the Elderly, a Concerted Action, SENECA; 23%) [48], the UK (National Diet and Nutrition survey; NDNS; 11%–27% free living, 30%–65% institutionalised) [49], and the US (National Health and Nutrition Examination Survey, NHANES; 15%–23% males, 14%–49% females) [50] This deficiency in older age has been attributed to increased requirements as a result of reduced absorption, increased catabolism and impaired phosphorylation as opposed to inadequate dietary intake [51,52]. 

Riboflavin deficiency is thought to mainly arise from inadequate dietary intake, particularly in those who do not consume dairy products or fortified foods [17,27,53]. A high prevalence of riboflavin deficiency is acknowledged in the developing world; less well recognised is the emerging evidence to suggest that sub-optimal status is also evident in developed countries. To date, most population based surveys only report dietary intake data, and relatively few include biomarker data. Despite dietary intakes being reported to be sufficient in the NDNS (5% below reference nutrient intake, RNI) [54] and in the Irish National Adult Nutrition Survey (NANS, 13% below EAR) [55], 39%–43% of older people had biochemical deficiency in the NDNS and 19%–22% in NANS. Thus, further population based surveys are still required to investigate the intake and requirements of older people based on robust biomarker data of both vitamin B6 and riboflavin. 

## 4. Assessment of B-Vitamin Biomarker Status

There are a number of direct and functional biomarkers available to determine B-vitamin status each with various strengths and limitations (Table 2). Plasma homocysteine accumulates with folate deficiency, it has been used as a biomarker of status but it lacks specificity as it can also be elevated by other B-vitamin deficiencies, including vitamins B12 [56], B6 [57] and riboflavin [58]. Serum folate is the earliest indicator of altered folate exposure and reflects recent dietary intake [59]. Red blood cell (RBC) folate is a sensitive indicator of long term folate status (during the preceding 120 days) [60,61]. It parallels liver concentrations this is considered to reflect tissue folate stores [20,62]. One recent meta-analysis demonstrated that both serum folate (27 RCTs) and RBC folate (12 RCTs) respond to interventions with folic acid in a dose-dependent manner and concluded that both indicators were robust measures of folate status [63]. To date, there is no gold standard biomarker for the assessment of vitamin B12 status despite there being a number of direct and functional measures available. Serum B12, although still widely used both clinically and in research, measures the total amount of the vitamin, however only 20% of this is metabolically active [64]. Serum total vitamin B12 can under-report the true prevalence of vitamin B12 deficiency [65,66] and up to 40% of older people can have low serum vitamin B12 but normal metabolic status [41,67]. Holo-transcobalamin (holoTC) measures the biologically active fraction of vitamin B12 and is considered to have superior diagnostic value to total B12 [68,69] although it can be affected by inborn errors altering intracellular vitamin B12 metabolism [68]. Plasma homocysteine can be used as a functional measure of vitamin B12 status but as previously discussed it is not specific to vitamin B12 (Table 2). Methylmalonic acid (MMA) is a more specific and sensitive functional biomarker of vitamin B12 status [31], the conversion of methylmalonyl-CoA to succinyl-CoA is a B12 dependant process, impaired B12 status leads to an accumulation of methymalonyl-CoA which then is metabolised to MMA and excreted in the urine. However, MMA is also elevated in renal dysfunction, which is common in older people [69] and so limits its use. Two reviews have recommended that at least 2 biomarkers are required in the diagnosis of vitamin B12 deficiency [42,70]. 

Plasma PLP concentration is the most widely used measure of vitamin B6 [71] with good specificity [72]. It is considered to reflect PLP concentrations in the liver [73]. Other markers of B6 are available including 4-pyridoxic acid and erythrocyte pyridoxal-5-phosphate. Erythrocyte glutathione reductase activation (EGRac) assay is the most widely used functional assay for riboflavin status and is generally regarded as the gold standard. EGRac measures glutathione reductase activity in erythrocytes before and after reactivation with its prosthetic group flavin adenine dinucleotide (FAD). A systematic review (14 RCTs) concluded that EGRac was a sensitive biomarker of change in riboflavin intake in populations with status ranging from deficient to normal [74].

In general, the use of different biomarkers or different cut off points for defining deficiency/suboptimal status when assessing individual B-vitamins can influence the interpretation and conclusion of relevant studies. This can make direct comparisons among studies difficult and may explain some inconsistencies in the literature as regards the role of B-vitamins in diseases in ageing. 

## 5. Consequences of B-Vitamin Deficiency

There are established clinical signs of B-vitamin deficiency (Table 3). The haematological manifestation of folate and vitamin B12 deficiency is indistinguishable, as both vitamins are linked through the enzyme methionine synthase [9] which catalyses the remethylation of homocysteine to methionine and thus are metabolically interrelated [79]. Deficiency of either vitamin results in a reduction of the active form of folate which subsequently results in megaloblastic anaemia [80], characterised by megaloblasts in the bone marrow, macrocytes in the peripheral blood and gigantism in the morphology of proliferating cells [25]. Vitamin B12 deficiency can also result in diverse neurological symptoms including irreversible nerve damage and sub-acute combined degeneration of the spinal cord if left untreated, as patients are often asymptomatic [81]. Neuropathy is quite specific to vitamin B12 and does not occur in folate deficiency [79]. Although severe vitamin B6 deficiency is relatively uncommon, it can present with notable symptoms such as anaemia, depression and sores or ulcers of the mouth [22]. The classical signs of riboflavin deficiency are angular stomatitis, cheilosis and glossitis, but these are rarely encountered in isolation and may be as a result of other B-vitamin deficiencies [27]. 

Apart from clinical deficiency signs, deficient or low status of B-vitamins can be associated with various adverse health outcomes throughout the lifecycle [82]. It should be noted that these can arise in the absence of more classical deficiency signs, and can occur within the range of what may be classed as “normal” within the clinical setting. 

## 6. Emerging Roles of B-Vitamin Status in Preventing Diseases of Ageing

### 6.1. Cardiovascular Health in Ageing

Clinical evidence has linked elevated plasma homocysteine concentrations and/or low folate with an increased risk of CVD. Early meta-analyses of observational studies concluded that lowering homocysteine would reduce the risk of heart disease by 11%–16% [83,84]. Homocysteine may however be a marker of suboptimal B-vitamin status and thus simply reflect a perturbation in one-carbon metabolism, rather than playing a causative role in CVD, but this is not universally accepted [90]. In any case, several secondary prevention randomised controlled trials (RCTs) published between 2004 and 2012 have failed to demonstrate a benefit of homocysteine-lowering by B-vitamin supplementation on CVD events generally [91,92,93,94,95,96,97]. These studies, however, typically involved patients with existing optimal B-vitamin status and/or advanced CVD, and thus a significant effect of B-vitamin supplementation on CVD risk may have been unlikely. 

The evidence however is generally much stronger for stroke than heart disease, with meta-analyses of earlier observational studies concluding that lowering homocysteine would reduce the risk of stroke by 19%–24% [83,84]. Furthermore, population data from the USA and Canada, reported an improvement in stroke mortality corresponding to the time that mandatory food fortification was introduced; in contrast, no similar improvement was found over the same time period in England and Wales where no mandatory fortification policy exists [98]. Of greatest relevance however are the findings of RCTs in relation to stroke risk, with meta-analyses of folic acid interventions showing a reduced risk of stroke by 18% overall [99], but with much greater reductions in risk noted in studies with longer folic acid treatment duration, with greater homocysteine reduction and in particular in those with no history of stroke [99,100]. More recent evidence including the China Stroke Primary Prevention Trial (CSPPT) [101] and a meta-analysis of 30 RCTs [102] also support a significant beneficial effect of folic acid supplementation on CVD risk (especially stroke), particularly in those with lower baseline folate status and without pre-existing CVD. Thus, optimisation of folate and the related B-vitamins might be beneficial in lowering CVD risk, particularly stroke, and most convincingly in primary prevention. 

In addition, genetic studies are now providing stronger evidence for the potential role of folate and the related B-vitamins in CVD, primarily through the investigation of the common 677 C → T polymorphism in the gene encoding the folate-metabolising enzyme MTHFR. Epidemiological evidence suggests that this common polymorphism increases the risk of CVD, especially stroke by up to 40% overall [84,103,104,105]. Furthermore, evidence is emerging to suggest that the excess genetic risk of CVD may be driven by higher blood pressure (rather than higher homocysteine), with meta-analyses of observational studies showing that the *MTHFR* 677 C → T polymorphism increases the risk of hypertension by 36%–87% [106,107]. This is also supported by recent evidence from a large study of Irish adults (*n* = 6069), which estimated that the *MTHFR* 677TT genotype was associated with an almost two-fold increased hypertension risk, a risk which was further increased when the genotype occurred in combination with low riboflavin status [15]. In addition, intervention studies from this centre, conducted in participants with premature CVD or hypertension showed that those with the *MTHFR* 677TT genotype were highly responsive to blood-pressure lowering through riboflavin supplementation, whilst no response was observed in participants with CC or CT genotypes [108,109,110]. Therefore, sub-populations worldwide with the *MTHFR* 677TT genotype (ranging from 3% to 32%) [14], may particularly be at greater risk of CVD via a blood pressure effect, and could benefit from a more optimal riboflavin status ideally before hypertension has developed.

### 6.2. Bone Health in Ageing

The role of B-vitamins in bone health has been extensively reviewed [16]. Briefly, observational evidence in older people has found independent associations of elevated homocysteine with bone mineral density (BMD) [111,112] and fracture risk [89,113]. Meta-analyses of observational studies have confirmed these relationships, with one (*n* = 14,863) concluding that homocysteine was an independent risk factor [114]. Another dose response meta-analysis (*n* = 11,511) estimated a 4% increased fracture risk for every 1 µmol/L increase of homocysteine concentration [115]. Low BMD has also been associated with sub-optimal status or dietary intakes of folate [111,116] or vitamin B12 [112,117]. Likewise, studies have also found an increased fracture risk in older people with sub-optimal status/intake of folate [118], vitamin B12 [119,120], or vitamin B6 [121,122]. Only one study to date in coeliac patients (*n* = 110) has examined riboflavin biomarker status with bone health (BMD), and found no relationship [16]. Although, low riboflavin intake in women (aged ≥55 years, *n* = 5035) was associated with a 1.8 times increased risk of osteoporotic facture and 2.6 times increased risk of fragility fractures [119]. The evidence is not entirely consistent, however, as some observational studies have shown no relationships with B-vitamin biomarkers on BMD [123] or fracture risk [124]. To date, there is limited RCT evidence linking B-vitamins with bone health and disease. One notable two-year RCT of combined folic acid and vitamin B12 supplementation (*n* = 628) resulted in a 75% reduction in the risk of hip fractures in older post-stroke Japanese patients, with low baseline folate status [125]. Certain RCTs with B-vitamins, which are not designed to examine bone outcomes (but rather other health outcomes), have reported no significant associations with fracture risk [126,127]. Additionally, interventions involving participants with generally higher baseline folate status have also reported no significant associations with BMD [128] or fracture risk [129]. This suggests that the benefit of interventions with B-vitamins may be confined to at-risk groups such as those with sub-optimal status. 

Genetic studies also support the potential role of folate and the related B-vitamins in bone health. The potential influence of *MTHFR* 677TT polymorphism on bone health may be mediated through impaired DNA methylation as a result of impaired DNA structure and stability [130,131], expression and the silencing of genes [132,133,134]. Epidemiological studies have reported that the *MTHFR* 677TT genotype is associated with significantly lower BMD [135,136] and a 2- to 2.5-fold increased risk of fractures [135,137]. This is also strengthened by one meta-analysis (3525 cases and 17,909 controls) which concluded that the *MTHFR* 677 C → T polymorphism was associated with BMD at multiple sites and individuals with the TT genotype had a 23% increased risk of all fractures [138]. Furthermore, the association appears to be influenced by prevailing B-vitamin intake, an interaction between the *MTHFR* 677TT genotype and low intakes of folate or riboflavin has been linked with lower BMD [139,140,141], higher bone loss and up to a three-fold increased fracture risk [118,142], although the majority of these studies do not consider B-vitamin biomarker status. This suggests that individuals with the *MTHFR* 677TT genotype and low B-vitamin status may be most vulnerable to poor bone health in later life. 

### 6.3. Brain Health in Ageing

#### Cognitive Dysfunction

Cognitive dysfunction ranges from mild cognitive impairment (MCI) to dementia and can result in a progressive loss of a number of specific cognitive functions [143,144]. The rate of cognitive decline varies between individuals [145], and it is estimated that 50% of older people with MCI will go on to develop dementia within five years of diagnosis [146]. Dementia has a number of distinct pathologies, although mixed pathologies in individuals have been shown at brain autopsies [147,148]. These include reduced/blocked flow to the brain [149], neuronal loss and damage to the connection between neurones [150] and the presence of Lewy bodies which has also been associated with neurotransmitter depletion [151]. Extra-neuronal β amyloid plaques and intra-neuronal neurofibrillary tangles [152] can also be present. Brain atrophy is a normal part of the ageing process, but with dementia, the rate is significantly higher overall and particularly in the hippocampus region [153,154]. Grey and white matter loss, thinning of cortical gyri and enlarged ventricles can also be evident in those with dementia [155]. 

Prospective studies make a strong case for elevated homocysteine and/or low B-vitamin status as potential causative factors in cognitive decline [156,157] and dementia [85]. This is further supported by three recent reviews and meta-analyses [87,158,159]. Epidemiological studies have also investigated the role of the relevant B-vitamins, with the focus mainly on folate and vitamin B12. Studies involving populations with lower baseline folate status in general support the role of folate in cognitive dysfunction [160,161,162] and cognitive decline [163,164], whereas those in countries with food fortification policies and thus overall higher mean concentrations of folate, are generally less supportive for a role in cognitive dysfunction [165,166], cognitive decline [167,168,169] or dementia [170]. Similarly, a number of large cohort studies have associated low vitamin B12 status (using more sensitive biomarkers of status including MMA and HoloTC) with cognitive dysfunction [160,166,171] and cognitive decline [162,172,173] in older people. However, the evidence for B12 is less convincing, and one meta-analysis has shown no association of vitamin B12 with cognitive decline or dementia (*n* = 14,325) [174]. This lack of significant association for B12 may be explained the fact that some of the included studies had methodological shortcomings (including limitations in the biomarkers used to determine B12 status). Thus, when sub-analysis of the data was carried out based on studies using more sensitive biomarkers of vitamin B12 status the results indicated that low vitamin B12 was in fact significantly associated with an increased risk of cognitive decline and dementia [174]. Relatively few studies have examined the role of vitamin B6 in cognition and even fewer have examined riboflavin. Low status of vitamin B6 has been shown to contribute to cognitive dysfunction [175,176] and cognitive decline [177]. Low vitamin B6 intake has been associated with a reduced risk of Alzheimer’s disease [178] in older Americans. Only one study from Taiwan has investigated the role of riboflavin status and found a significant association with cognitive dysfunction, which was assessed using only a short portable mental status questionnaire and cognition was not the primary outcome [179]. 

Several RCTs have investigated the potential role of folate alone or in combination, with vitamins B12 and/or B6 in maintaining cognitive health in ageing, although to date most include high pharmacological doses and none include riboflavin (Table 4). One meta-analysis of nine RCTs in healthy people (*n* = 2835) concluded that folic acid had no effect on cognitive function [180]. However, in this meta-analysis, only two studies had more than 275 participants and were longer than 12 months in duration (five were ≤ 6 months). Additionally, one recent controversial meta-analysis in almost 22,000 healthy older people concluded that neither folic acid nor vitamin B12 had a beneficial effect on “cognitive ageing” [181]. The results have been widely criticised, mainly due to the inclusion criteria used to select the trials [182,183,184]. Only three of the RCTs had final cognitive test scores and two thirds of the baseline results were not available. MMSE, a screening tool that is considered as a crude measure of global cognition, was the main cognitive assessment tool used. Furthermore, this meta-analysis did not identify subgroups or at risk groups in each trial that may have benefited from B-vitamins. The recent B-Vitamins for the Prevention of Osteoporotic Fractures (B-PROOF) study did show a slower rate of global cognitive decline, this was attributed to chance by the authors [185]. In general, greatest cognitive benefits have been demonstrated in RCTs involving participants with lower baseline folate status [186,187], higher homocysteine concentrations [188,189] and lower B-vitamin intake [190]. In contrast, RCTs in individuals with higher baseline folate status [191] or higher cognitive status [185] have shown no significant benefit on cognitive performance. This supports the view that baseline B-vitamin status is a critical consideration in the outcome of trials examining cognition, and further is supported by a systematic review involving 14 RCTs, which found no overall benefit of B-vitamin interventions, but reported a benefit in cognitive function in those with lower baseline folate status [192]. Thus, well designed RCTs are still required, particularly in those with sub-optimal B-vitamin status as they might benefit most in optimising B-vitamin status to maintain cognitive health in ageing.

Apart from evidence from observational studies and RCTs, genetic studies also provide support for the role of B-vitamins in maintaining cognitive health in ageing. The *MTHFR* 677TT genotype has been associated with poorer cognitive performance in Chinese males [197] and a 42% increased risk of cognitive impairment in older Australian males [198], but not in older Americans [166]. Meta-analyses have also associated the *MTHFR* C677T polymorphism with an increased risk of Alzheimer’s disease [199], and dementia albeit in Asian populations only [200,201]. These results may be attributed to the varying prevalence of the *MTHFR* 677TT genotype worldwide and the modulating effect of B-vitamin status. To date, no studies have investigated interactions between this gene and B-vitamin status in relation to cognitive health. 

### 6.4. Future Directions

While questionnaire based tools tests are typically used to assess cognitive performance in observational studies and RCTs, they do have inherent limitations. The use of brain imaging technology is considered a more robust measure of cognitive and brain health as it can overcome the inherent weaknesses of cognitive questionnaire based tools. MRI scans have shown that vitamin B12 status is inversely related to the rate of overall brain atrophy [202], and lower B12 status has been associated with a reduced microstructure integrity of the hippocampus [203], increased rate of brain volume losses [204,205], and a greater severity of white matter lesions [206]. Likewise, lower folate status has also been associated with severe cortical and hippocampal brain atrophy [207] and white matter lesions [161]. Furthermore, the VITACOG trial demonstrated that combined folate, B12 and B6 supplementation over two years markedly reduced (by up to 53%) the rate of brain atrophy. This effect was modulated by baseline homocysteine concentration, with the greatest impact observed in those with concentrations ≥ 13 µmol/L [196]. A reduction in cerebral atrophy by as much as seven fold was also observed in grey matter regions, which are the most relevant regions for Alzheimer’s disease pathology [188]. More recently, the use of a new functional neuroimaging technique, magnetoencephalography (MEG), has emerged which can map brain activity and assess cognitive processes and specific functions of the brain [208]. MEG is currently being used in some clinical studies, including cognitive studies in ageing, but to date none have examined B-vitamins. Brain imaging could therefore help further elucidate the role and underlying mechanism linking B-vitamins with brain health and disease. 

Future studies are therefore warranted to investigate the effects (and interactions) of folate, the related B-vitamins, and relevant genetic variants, particularly in cohorts with lower B-vitamin status. Longitudinal studies are also required to explore differential effects on cognition over time. Targeted RCTs of sufficient duration, with lower doses of B-vitamins at levels achievable through dietary means, are also indicated. 

## 7. Potential Mechanisms Linking B-Vitamins with Diseases of Ageing

Although no clear mechanisms have been established, a variety of biologically plausible mechanisms have been suggested to explain the role of B-vitamins in the diseases of ageing [85,156]. In the brain and other tissues, folate and the metabolically related B-vitamins are required as co-factors for one-carbon transfer reactions, which in turn are fundamental for the synthesis of DNA and RNA nucleotides, the metabolism of amino acids and the maintenance of methylation reactions [6,7]. Thus, the proposed mechanisms by which sub-optimal B-vitamins status or deficiency could contribute to greater risk of cognitive impairment and other diseases of ageing, involve perturbations in the complex regulatory network maintaining one-carbon metabolism [11,12]. This can result in hyperhomocysteinemia [209] and/or reduced methylation status within the relevant tissue [210,211], increased misincorporation of uracil into DNA, and altered RNA [212,213,214] and neurotransmitter products [215]. Vitamin B6 is important in brain health as pyridoxine is a co-factor in transamination and decarboxylation reactions required for the metabolism of several neurotransmitters, including serotonin, dopamine, and histamine [216]. Elevated homocysteine and/or lower B-vitamins can also impact on the immune system and cause increased inflammation and antioxidant damage, alone or in synergy, which can have adverse effects on the disease of ageing through the vascular system and atherosclerosis [217,218,219]. B-vitamins have also been shown to have an indirect role on bone remodelling and protective effects on bone formation which help to maintain bone health in ageing [220,221,222]. Cognitive deficits and Alzheimer’s disease has also been observed in B-vitamin deficient rats and been attributed to abnormal methylation [223,224]. 

Specific genetic polymorphisms, including the common *MTHFR* 677 C → T polymorphism can disrupt normal one-carbon metabolism and thus have an impact on diseases of ageing, independent of homocysteine, as a result of impairments in methylation activity; improved B-vitamin status could help to modulate this risk [13,225,226]. Furthermore, this polymorphism has more recently been associated with blood pressure while riboflavin has emerged as having a novel role in lowering blood pressure in hypertensive patients with the variant *MTHFR* 677TT genotype [110]. The effect of the *MTHFR* 677 C → T polymorphism on blood pressure might be mediated by nitric oxide, a potent vasodilator, and riboflavin has been demonstrated as a means to restoring MTHFR activity in vivo [58]. The interaction between the *MTHFR* genotype, B-vitamin status and risk of hypertension requires further exploration. 

## 8. Public Health Implications

Folic acid food fortification was instigated primarily to reduce the prevalence of neural tube defects (NTDs) but it may also have additional benefits for the ageing population. Currently, 80 countries have mandatory folic acid food fortification policies in place, including America and Canada [227]. As a result of fortification, there has been a dramatic fall in NTDs [228] and folate deficiency in the USA and Canada is practically non-existent [34,36,229]. Mandatory folic acid food fortification has also lead to an improvement in stroke mortality in the US and Canada [98] and could have additional beneficial effects in maintaining better cognitive function [230] and bone health [231] in older people. No European country has a mandatory folic acid food fortification policy but most do allow voluntary food fortification. The UK and Ireland have quite liberal voluntary fortification polices and are currently considering mandatory fortification. Voluntary food fortification has resulted in significantly higher dietary intakes and biomarker status of folate and related B-vitamins in Irish adults of all ages [232,233]. The NANS dietary survey however highlighted that the overall 21% of Irish adults who were non-consumers of fortified foods were at higher risk of sub-optimal status of folate and related B-vitamins [233]. Regular fortified food consumption therefore has the potential to improve dietary and biomarker status of B-vitamins but a mandatory food fortification would ensure that the whole population were protected. 

Low vitamin B12 status in older people is also a concern. Current dietary recommendations for vitamin B12 range between 2.4 μg/day in the USA [234] and 4 μg/day in Europe [235]. However, the European recommendations do not account for the high prevalence of food-bound malabsorption in older people, whereas people aged ≥50 years in the USA are recommended to consume most of their vitamin B12 from crystalline sources (i.e., fortified food and supplements) in order to overcome food-bound B12 malabsorption [234]. The synthetic form of vitamin B12, found in fortified foods and supplements, is freely available and thus has no gastric acid requirement [40]. Furthermore, there was a previous safety concern that the high folic acid intake may potentially mask the anaemia of vitamin B12 deficiency and thus delay diagnosis resulting in irreversible nerve damage [79,236]. Mandatory food fortification in the USA however has not led to these adverse events [237,238,239]. Therefore, from a public health perspective, there are significant challenges in relation to maintaining vitamin B12 in older people and vitamin B12 food fortification could help address some of these challenges. 

Concerns have been raised as regards a possible adverse interaction of high folate with low vitamin B12 leading to an increased risk of cognitive impairment in the USA and Australia [230,240,241], but this is rather controversial. Two studies in the UK and USA failed to detect such an adverse interaction [238,242] and one study in the Netherlands in fact found that this combination (i.e., high folate and low B12) was associated with a reduced risk of cognitive impairment [243]. Discrepancies among studies in this regard may in part be attributed to the relatively low numbers in most studies with this folate/vitamin B12 combination and the type of biomarker used or the cut off value used to define low status in each study. Additionally, a recent US review concluded that the current observational data was limited and that no RCTs had investigated this folate–B12 interaction in cognitive health [244]. Concerns have also been raised that excessively high folate intakes could have cancer promoting effects in segments of the ageing population [245,246,247]. A Scientific Advisory Committee on Nutrition (SACN) review in the UK however found insufficient evidence to support the view that mandatory folic acid fortification would promote cancer [248]. Excessive folic acid intakes have also been reported to increase un-metabolized folic acid in the circulation with potential adverse effects on health [34,249], although a recent international review did not find any health concerns even at very high folic acid levels [20]. Suggestions have been made to include both folic acid and vitamin B12 in mandatory food fortification policies, to prevent adverse health outcomes and address food-bound malabsorption but the costs of such an approach may be prohibitive. Furthermore, vitamin B6 and riboflavin status in older people is also of concern and may be much more important than previously appreciated for public health in ageing. Therefore, those contemplating public health issues worldwide need to consider a balanced approach and should endeavour to achieve optimal status of all relevant B-vitamins throughout all stages of life. 

## 9. Conclusions

In summary, folate and the metabolically related B-vitamins, B12, B6 and riboflavin, have a vital role in maintaining one-carbon metabolism and any perturbation in this pathway as a result of low/deficient B-vitamin status can impact on health through a number of related mechanisms. Evidence is accumulating to support the beneficial role of B-vitamins in maintaining cardiovascular, bone and brain health in ageing, with the most at risk sub-populations benefiting from optimising one/more B-vitamins involved in one-carbon metabolism. To date, studies investigating the role of B-vitamins in health and disease have tended to focus predominantly on folate and vitamin B12. Future well designed research is warranted to further investigate the metabolically linked B-vitamins B6 and riboflavin, and should include robust and sensitive measures of B-vitamins as well as novel measures of the health outcome of interest (e.g., imaging techniques to assess brain function). In an era where personalised nutrition has gained much attention, further work is also needed to explore the role of the interaction of the *MTHFR* 677 C → T polymorphism with both folate and riboflavin, given that this gene–nutrient interaction has the potential to modulate the risk of disease.

## Figures and Tables

**Table 1:** Causes of B vitamin deficiency.

B Vitamin	Inadequate Intake	Increased Requirement	Malabsorption	Drug–Nutrient Interactions	Other
**Folate** [19,20,21,22,23,24]	Common Poor cooking techniques	Elderly Pathological conditions	Intestinal diseases Coeliac disease Crohn’s disease Ulcerative Colitis	Phenytoin Phenobarbital/Primidone Trimethoprim Methotrexate Sulfasalazine Metformin	Alcohol abuse Genetic disorders Haemolytic anaemia
**B12** [19,21,22,25,26]	Common Vegan diets	Elderly	Intestinal diseases Coeliac disease Crohn’s disease Gastric/intestinal resection Atrophic gastritis Bacterial overgrowth Helicobacter pylori Pancreatic insufficiency Pernicious anaemia Zollinger–Ellison Syndrome	Proton pump inhibitors H2-receptor antagonists Metformin Nitrous oxide Colchicine	Alcohol abuse Genetic disorders Tropical or non-tropical sprue
**B6** [21,22,25,26]	Rare Chronic dieters	Elderly	HIV	Isoniazid Anti-Convulsants Steroids	Alcohol abuse Genetic disorders Liver disease Renal dialysis Rheumatoid arthritis
**B2** [21,25,26,27]	Common Chronic dieters	Elderly	Diabetes Liver disease Thyroid and renal insufficiency GI and biliary obstruction	Phenothiazines, e.g., chlorpromazine Theophylline	Alcohol abuse Genetic disorders Hypochromic anaemia Metals such as zinc, copper and iron

**Table 2:** Assessment of B-vitamin biomarker status.

		Biomarker	Strengths	Limitations
**Homocysteine** [20]	**Functional**	Plasma homocysteine	Sensitive functional biomarker Highly responsive to intervention with B-vitamins Responds within 3–4 weeks of B-vitamin depletion and subsequent repletion Very stable analyte Can be stored frozen for extended periods of time	Lacks specificity as affected by other B-vitamins Requires separation from RBCs within one hour of blood collection, or <8 h if whole blood is kept on ice Influenced by other factors such as lifestyle, genetics, renal insufficiency, age and medications
**Folate** [20,21,60,63,75]	**Direct**	Serum/Plasma folate	Earliest indicator of altered folate exposure Reflects recent dietary folate intake Requires less time processing at time of blood collection vs. RBC folate Can be measured in the field	Inconsistent use of cut off values makes comparisons across different methods and labs difficult Fasting blood samples are recommended
		Red cell folate	Sensitive indicator of long-term folate status Reflects folate status over half-life of RBCs Reflects tissue folate stores as parallels liver concentrations Highly correlated with habitual intake when expressed as DFEs	Affected by vitamin B12 deficiency Inconsistent use of cut off values makes comparisons across different methods and labs difficult Cannot be measured in the field
**B12** [21,34,60,70,75,76]	**Direct**	Serum/Plasma total B12	Serum standard clinical test Variety of assays available Measures all forms of vitamin B12	Does not reflect intracellular vitamin B12 Falsely elevated B12 caused by factors including liver disorders, bacterial overgrowth, renal failure Falsely low B12 caused by factors such as iron deficiency, HIV infection and pregnancy Inconsistent use of cut off values makes comparisons across different methods and labs difficult
		Serum/Plasma Holo-transcobalamin (HoloTC)	Represents metabolically active fraction of B12 Decrease in holoTC can indicate earliest sign of B12 depletion Considered better indicator of B12 status in elderly	Highly sensitive to altered renal function and influenced by factors including genetics
	**Functional**	Serum/plasma/urine Methylmalonic acid (MMA)	Reflects availability of intracellular B12 Early detection of functional B12 deficiency Not affected by folate deficiency	Lacks sensitivity as can be elevated in those with renal impairment High running costs
**B6** [21,60,72,77,78]	**Direct**	Plasma Pyridoxal-Phosphate (PLP)	Most widely used Good specificity and reflects PLP content in liver Responds quickly within 1–2 weeks of B6 depletion and subsequent repletion Reference ranges available for younger and older adults Fairly stable at low temperatures	Does not represent PLP content in the muscle which is resistant to B6 depletion Influenced by other factors such as age, sex, pregnancy, protein and alcohol intake PLP declines in samples stored at room temperature and exposure to light Fasting blood samples are recommended Plasma PLP concentrations affected by use of certain drugs
		Erythrocyte PLP	Positively correlated with B6 dietary intake Responds within weeks of B6 depletion and subsequent repletion Appears more responsive than plasma PLP to supplementation May be more reliable marker than plasma PLP under conditions and disease associated with inflammation	Affected by haemoglobin variants Assay is cumbersome, with variable recovery and low precision
**B2** [21,60,74,78]	**Direct**	Serum/Plasma/Erythrocyte Riboflavin/Flavine Adenine Dinucleotide (FAD)/Flavin Mononucleotide (FMN)	Riboflavin vitamers are stable for several years when plasma samples are stored at −80 ° CSerum/plasma can be used retrospectively in a hospital setting	Influenced by other factors such as age, sex, pregnancy, protein and alcohol intake Serum/plasma riboflavin concentrations affected by use of certain drugs High variability within and between-subjects compared to the cofactor forms of riboflavin (plasma/erythrocyte)
	**Functional**	Erythrocyte glutathione reductase activation (EGRac) assay	Most widely used marker of status Measures tissue saturation and long term status Enzyme is stable for several years when erythrocyte lysates are stored at −80 °C	Poor index of optimum riboflavin status Assay is not linear against status Difficult make comparisons across different methods and labs

**Table 3:** Consequences of deficient or low status of B-vitamins.

	Clinical Deficiency Signs [19,20,21,22]
**Folate**	Megaloblastic anaemia, clinical features characterised by megaloblasts in the bone marrow macrocytes in the peripheral blood gigantism in the morphology of proliferating cells
**B12**	Megaloblastic anaemia indistinguishable from folate-related megaloblastic anaemia Irreversible nerve damage/neuropathy Sub-acute combined degeneration of the spinal cord (SCD)
**B6**	Notable symptoms include: Microcytic anaemia Inflammation of the tongue Sores or ulcers of the mouth Dermatitis Nervous/muscular signs Irritability, fatigue, numbness Headache, muscle twitching Difficulty walking, convulsions Depression and confusion
**B2**	Classic signs arbioflavinosis, rarely encountered in isolation Anaemia Cheliosis, Angular stomatitis Glossitis Redness and swelling of the lining of the mouth and throat Seborrheic dermatitis particularly affecting the nose, cheeks and forehead Eyes burning and itching Sensitivity to light Loss of visual acuity Gritty sensation under the eyelids
	**Health Consequences of Low Status of Folate and/or Other B-Vitamins [83,84,85,86,87,88,89]**
	Elevated homocysteine CVD and stroke Cognitive decline/dementia/Alzheimer’s Osteoporosis and risk of fractures

**Table 4:** Summary of Randomised Controlled Trials of 2 years or more assessing the effect of B vitamins on cognitive function in ageing.

Author/Year/Trial	Country	Sample Size (*n*)	Age (Years)	Population Studied Plasma tHcy (µmol/L)	Treatment (mg/day)	Duration	Cognitive Outcomes
***Questionnaire based assessment***							
McMahon 2006 [191]	New Zealand	276	≥65	Healthy tHcy > 13	1.0 FA, 0.5 B12, 10 B6	2 years	No significant effect on cognition
**FACIT** Durga 2007 [186]	The Netherlands	818	50–70	Healthy tHcy 13–26	0.8 FA or placebo	3 years	Improvement in domains including memory, information-processing and sensorimotor speed No improvement in global cognition or domains of complex speed or word fluency
**WAFACS** Kang 2008 [190]	USA	2009	≥65	CVD/high risk women tHcy not provided	2.5 FA, 1.0 B12, 50 B6 or placebo	6.6 years	Reduced risk of cognitive decline among women with low baseline dietary intake of B-vitamins Overall no significant effect on rate of cognitive decline
Brady 2009 [193]	USA	659	Mean 67.3	Advanced renal disease tHcy ≥ 15	40 FA, 2.0 B12, 100 B6 or placebo	5 years	No significant effect on cognition
**Health in Men Study**: sub set Ford 2010 [194]	Australia	299	≥75	Hypertensive men Mean tHcy 13.1–14	2.0 FA, 0.4 B12, 25 B6 or placebo	2 years	No significant effect on cognition
Kwok 2011 [189]	Hong Kong	140	≥60	Dementia diagnosis Mean tHcy 14.1	5.0 FA, 1.0 B12 or placebo	2 years	Improvement in domain of construction No change in global cognitive decline, attention, memory or conceptualisation
**Beyond ageing project** Walker 2012 [187]	Australia	900	60–74	Elevated psychological distress Mean tHcy 9.7	0.4 FA, 0.1 B12 or placebo	2 years	Improvement in overall global cognition and in domains of immediate and delayed recall scores No significant change in other cognitive domains
**VITACOG** De Jager 2012 [195]	UK	168	≥70	MCI Mean tHcy 11.3	0.8 FA, 0.5 B12, 20 B6 or placebo	2 years	Slower decline in global cognition and in domains of semantic and episodic memory Clinical benefit in global clinical dementia rating score
**BPROOF** Van Der Zwaluw 2014 [185]	The Netherlands	2919	≥65	Healthy tHcy 12–50	0.4 FA, 0.5 B12, 0.15 D3 or placebo + D3	2 years	Slower rate of decline in global cognition No change in domains of memory
***Brain-imaging assessment***							
**VITACOG** Smith 2010 Douaud 2013 [188,196]	UK	168	≥70	MCI Mean tHcy 11.3	0.8 FA, 0.5 B12, 20 B6 or placebo	2 years	Slowed shrinkage of brain Marked reduction in cerebral atrophy in grey matter regions
**BPROOF** Van Der Zwaluw 2014 [185]	The Netherlands	2919	≥65	Healthy tHcy 12–50	0.4 FA, 0.5 B12, 0.15 D3 or placebo + D3	2 years	*Awaiting MRI scan results*

Abbreviations: tHcy, homocysteine; FA, folic acid; MMA, methylmalonic acid; holoTC, holo-transcobalamin; EPA, eicosapentaenoic; DHA, docosahexaenoic acid; MCI, mild cognitive impairment; MRI, magnetic resonance imaging; CVD, cardiovascular disease.
